# Six weeks of home enteral nutrition versus standard care after esophagectomy or total gastrectomy for cancer: study protocol for a randomized controlled trial

**DOI:** 10.1186/1745-6215-15-187

**Published:** 2014-05-24

**Authors:** David J Bowrey, Melanie Baker, Vanessa Halliday, Anne L Thomas, Ruth Pulikottil-Jacob, Karen Smith

**Affiliations:** 1 Department of Surgery, University Hospitals of Leicester NHS Trust, Level 6 Balmoral Building, Leicester Royal Infirmary, Leicester LE1 5WW, UK; 2 School of Health and Related Research, University of Sheffield, 30 Regent St, Sheffield S1 4DA, UK; 3 Department of Oncology, Clinical Sciences Building, University of Leicester, Leicester LE1 5WW, UK; 4 Department of Health Economics, Room A101, University of Warwick, Warwick CV4 7AL, UK; 5 Department of Health Sciences, University of Leicester, 22-28 Princess Rd West, Leicester LE1 6TP, UK

**Keywords:** Enteral nutrition, Feasibility, Gastrectomy, Gastric cancer, Jejunostomy, Qualitative, Esophageal cancer, Esophagectomy

## Abstract

**Background:**

Each year approximately 3000 patients in the United Kingdom undergo surgery for esophagogastric cancer. Jejunostomy feeding tubes, placed at the time of surgery for early postoperative nutrition, have been shown to have a positive impact on clinical outcomes in the short term. Whether feeding out of hospital is of benefit is unknown. Local experience has identified that between 15 and 20% of patients required ‘rescue’ jejunostomy feeding for nutritional problems and weight loss while at home. This weight loss and poor nutrition may contribute to the detrimental effect on the overall quality of life (QoL) reported in these patients.

**Methods/Design:**

This randomized pilot and feasibility study will provide preliminary information on the routine use of jejunostomy feeding after hospital discharge in terms of clinical benefits and QoL. Sixty participants undergoing esophagectomy or total gastrectomy will be randomized to receive either a planned program of six weeks of home jejunostomy feeding after discharge from hospital (intervention) or treatment-as-usual (control). The intention of this study is to inform a multi-centre randomized controlled trial. The primary outcome measures will be recruitment and retention rates at six weeks and six months. Secondary outcome measures will include disease specific and general QoL measures, nutritional parameters, total and oral nutritional intake, hospital readmission rates, and estimates of healthcare costs. Up to 20 participants will also be enrolled in a qualitative sub-study that will explore participants’ and carers’ experiences of home tube feeding.

The results will be disseminated by presentation at surgical, gastroenterological and dietetic meetings and publication in appropriate peer review journals. A patient-friendly lay summary will be made available on the University of Leicester and the University Hospitals of Leicester NHS Trust websites. The study has full ethical and institutional approval and started recruitment in July 2012.

**Trial registration:**

UKClinical Research Network ID #12447 (Main study); UKCRN ID#13361 (Qualitative sub study); ClinicalTrials.gov #NCT01870817 (First registered 28 May 2013)

## Background

Each year 13,500 patients in the United Kingdom are diagnosed with esophagogastric cancer [1]. Approximately a quarter of these patients will undergo surgery, with an ensuing median survival of around 2.5 years. Overall, after surgery 30% of patients will be cured of their cancer [2].

Nutritional support is considered important from diagnosis, through treatment, and in the palliative stages of the disease. Feeding tubes (jejunostomy (JEJ)) inserted into the small bowel during surgery allow early postoperative nutrition while patients are nil by mouth. JEJ are recommended by international groups [3] as they have been shown to have a positive impact on clinical outcomes in short-term studies [4-6]. Despite this, the proportion of patients having a feeding jejunostomy inserted varies greatly between NHS centers across the country [7] and the timing of discontinuing JEJ feeds is variable and not always related to the adequacy of oral nutritional intake [8]. The effect of supplementary JEJ feeds on oral intake has not been prospectively assessed but it is routine for tube feeds to be discontinued one to two weeks post-surgery in the belief that this will help promote a return to eating.

At University Hospitals of Leicester NHS Trust it is observed practice that 15 to 20% of patients require ‘rescue’ JEJ feeds to be re-instigated post-discharge due to failing nutrition. This can require readmission to hospital. The prevalence of home JEJ feeding after esophagogastric cancer surgery has been reported at between 15 and 48% in published studies that have employed home JEJ feeding [9-11]. It is unclear whether the practice of feeding all patients by JEJ at home would be beneficial. Studies that report medium-term endpoints are lacking and are required to provide evidence of the benefits of adjunctive nutrition support in this patient group

Few studies have assessed the potential benefits of continuing supplementary JEJ feeds after hospital discharge [12]. In an institutional review of practice [13] comparing the historical practice of not providing home enteral support with the recent practice of home jejunostomy feeding, home enteral support was associated with better weight maintenance. The effect of enteral nutrition on other outcome measures such as quality of life is not known.

Following esophagectomy or total gastrectomy patients often struggle to establish an adequate nutritional intake orally due to a combination of poor appetite, a fear of eating, early satiety, abnormal gut transit, gastrointestinal symptoms such as nausea, reflux, and altered bowel habits.

Studies have shown that over 60% of patients have an inadequate oral intake at time of hospital discharge, consuming 70 and 65% of their energy and protein requirements respectively [14]. An earlier study evaluating patients six months after esophagectomy demonstrated that 64% of patients reported a loss of greater than 10% of their baseline body mass index [15]. Estimates of the time taken to establish what would be considered a socially acceptable diet after surgery are around six months [16].

Weight loss and poor nutritional intake may contribute to the detrimental effect on overall quality of life (QoL) reported in these patients [17]. Although in general, tumor location and stage of disease are the major determinants of QoL, in esophageal cancer patient nutritional factors (dietary intake and weight loss) have been shown to be as important in determining functional QoL scores [18]. Global quality of life scores have been correlated with both postoperative BMI and weight loss [19].

Little is known about the patient experience of home tube feeding and studies often focus on heterogeneous populations of variable age and dependency [20]. In esophagogastric patients, the JEJ tube already remains in place but any additional effect of using it is not known. The effect on family and carers and their relationship to the patient must also be considered as home tube fed subjects are often reliant on support, and carers perceive QoL very differently to that of the patient [21,22]. This may increase the burden of treatment.

The aim of the current study is to pilot an investigation of the impact of six weeks of home JEJ feeding in patients undergoing esophagectomy or total gastrectomy for cancer and assess the feasibility of conducting an appropriately powered multi-centre trial to evaluate the effectiveness of six weeks of home JEJ feeding. Specifically this study will: (1) estimate participant recruitment and retention rates in order to inform a larger multi-centre trial, (2) estimate variability in disease-specific and general QoL measures, (3) explore the relationship between disease-specific and general QoL measures, (4) explore the effect of home feeding on nutritional parameters and total and/or oral intake, (5) estimate hospital readmission rates, (6) explore participants’ and their carers' experiences of living with a jejunostomy tube *in situ* and that of home tube feeding, (7) test the feasibility of estimating healthcare costs, (8) provide preliminary evaluation of the cost-effectiveness of postoperative home jejunostomy tube feeding compared with standard care from an NHS and personal social service.

## Methods/Design

### Study design and setting

This is a pilot prospective two-arm mixed-method randomized controlled trial (RCT), with treatment-as-usual as the control. Given the nature of the intervention and control it is not possible to blind participants or those responsible for patient care. To minimize bias the research dietitian who will collect study data will have no involvement in patient care. This study will be conducted at a single site, University Hospitals of Leicester NHS Trust, which draws patients from Leicester, Leicestershire and Northamptonshire.

### Trial management group

A trial management group has been setup including the investigators, representatives from the Leicester Clinical Trials Unit, and two former patients (one with their partner). This group will meet every six months for the duration of the study to review study progress.

### Participants

This study will recruit adult patients referred to the University Hospitals of Leicester NHS Trust Esophagogastric Cancer Service with a confirmed diagnosis of esophagogastric cancer, deemed suitable for an esophagectomy or total gastrectomy. The inclusion criteria are adults over 18 years of age with a confirmed diagnosis of esophagogastric cancer who are undergoing a planned curative surgical treatment esophagectomy (Ivor Lewis, three stage, or transhiatal resection) or total gastrectomy with placement of jejunostomy feeding tube. Patients will provide written informed consent for participation in the study.

The exclusion criteria include an inability to provide informed written consent, patients in whom artificial nutrition support at home is deemed inappropriate by either the patient or healthcare team (due to safety issues or home circumstances) and patients undergoing subtotal gastrectomy (would not usually have jejunostomy tube fitted).

Participation in this study does not exclude participation in national trials of perioperative chemotherapy. Due to the different scheduling arrangements the two treatment interventions (chemotherapy and jejunostomy feeding) would not be administered concurrently. The time frame for the administration of preoperative chemotherapy is prior to the planned six week jejunostomy feeding period, and the time frame for the administration of postoperative chemotherapy is after completion of the planned six week jejunostomy feeding. Some participants and their carer or partner will also participate in the qualitative study, for which the only additional criterion is a willingness to participate.

### Recruitment

Potential participants will be identified at the weekly multidisciplinary, upper gastrointestinal cancer meetings by a team member involved in the patient’s care. At the surgical clinic potential participants will asked by a member of the healthcare team whether they are happy to receive information about the study. If they agree they will be provided with a participant information leaflet either by a member of the research team (if available) or the clinical team, and asked to give their consent for a member of the research team to contact them by telephone. A minimum of 24 hours later a member of the research team will contact the potential participant by telephone. If they agree to take part they will be visited in hospital at their pre-assessment clinic appointment. Those patients who do not wish to be contacted by telephone can inform their clinician that they wish to take part or telephone the research team.

Potential participants for the qualitative exploratory study will be those recruited into the RCT and their carers. At the initial recruitment stage participants will be asked to indicate on the consent form if they agree to be interviewed. Following the six-week intervention period, potential participants will be contacted and invited to be interviewed. Where a partner or main carer is identified, they too will be asked if they are interested in participating. On agreement, a volunteer information sheet will be posted out and consent will be taken prior to the interview taking place. It is envisaged that including the partner or carer will add to the depth of information, particularly regarding experiences of home tube feeding. The sample will also include male and female patients without family or support at home. Participants will be given the option to be visited at home or to attend the hospital for interview. Recruitment to the study will be conducted over a 20-month-period and individual participation in the study will last for 7 months.

### Sample size

Home JEJ feeding is not routinely carried out at present and there is no published data on the variables of interest in this patient population. Consequently the sample size in this pilot study was chosen to enable a sensible estimation of the quantities of interest, in particular variability, whilst not exposing too large a number of participants to the full range of experimental procedures.

We therefore aim to recruit 60 subjects, 30 randomized to home JEJ and 30 to treatment-as-usual, which (allowing for up to 17% early withdrawal) would result in 50 participants completing the 6-week intervention period.

### Randomization

Participants are randomized to either the control or intervention group at enrolment, and prior to surgery, so that baseline QoL and nutritional parameters can be collected. We anticipate an early withdrawal rate due to perioperative mortality or morbidity of around 5% based on local experience. At the time of the operation it may not be clinically suitable to place a JEJ tube in all subjects, or some will not have a functioning JEJ tube (approximately 2%, due to kinking or blockage).

The randomized schedule is managed by the University of Leicester Clinical Trials Unit, using computer generated random assignment, using permuted block randomization, stratified for type of procedure (esophagectomy or total gastrectomy). A member of the research team (dietitian or lead clinician) will randomize the participant through an electronic Interactive Web Response System (IWRS). Participants are sequentially assigned to groups as they are entered into the study and the IWRS will provide a trial participation number.

Neither the study participants nor the research team are blinded to the allocation group. However the research dietician has no role in determining clinical care unless there are patient safety concerns. The research team did not consider it either ethical or practical to administer placebo feed to the control group.

### Standard postoperative care

**Figure 1:**
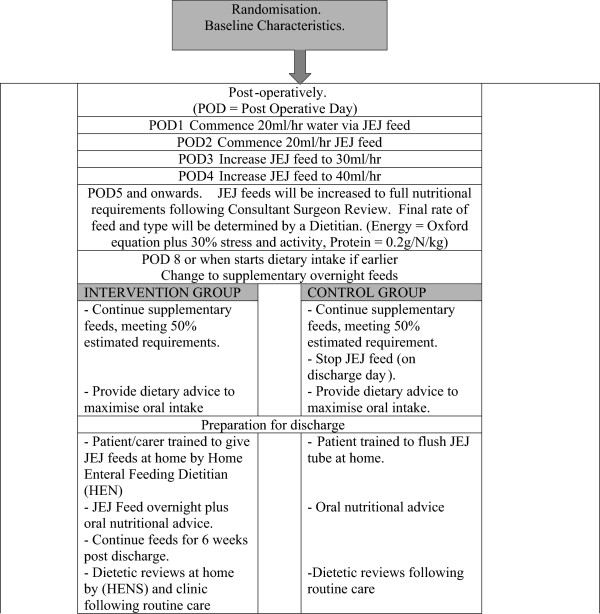
Plan for jejunostomy (JEJ) feeds in hospital (routine care) and post-discharge (intervention and control groups).

All participants will receive standard postoperative care whilst in hospital, consisting of feeds via a JEJ tube placed at the time of surgery. Tube insertion, commencement of feeds, and subsequent increase in volume will follow normal clinical care (Figure 1).

Continuous JEJ feeds will be reduced to supplementary overnight feeds (10 to 15 hours duration) when oral intake starts (at approximately postoperative day 7) and continue until the morning of the day of hospital discharge in all participants. Dietary advice, including food fortification and the use of prescribed nutritional supplements, with supporting written information will be provided to all subjects by the clinical team prior to discharge.

### Intervention

Participants will be referred to the Home Enteral Nutrition Service (HEN) and they will be taught (with or without family support) to independently manage the JEJ feed at home. For the first six weeks after discharge from hospital, the participant and/or carer will be trained to administer overnight jejunostomy feeds via an electronic feeding pump. The timings of feeds will be decided in conjunction with the participant’s normal and expected routine following discharge home. The participant’s individual nutritional requirements will be assessed using prediction equations [23,24] aiming to provide at least 50% of energy and protein needs via the JEJ feed. The final amount provided for each subject will be adjusted depending on the oral intake and weight change as assessed by the Home Enteral Nutrition Service Dietitian (HEN).

Post-discharge dietetic follow-up will involve a routine telephone call and home visit from HENs within the first week of discharge and additional HEN input will be dictated by nutritional need but will include at least one further contact within the six-week intervention period. It may be necessary to reduce the rate and amount of JEJ feed the participant receives to effectively manage complications (gastrointestinal intolerance). The initial adjustments will be to reduce the feed rate by 10 – 25 ml/hr, keeping the total volume of feed delivered the same, but infused over a longer period (for example 100 ml/hr over 10 hours reduced to 80 ml/hr over 12.5 hours. The second step will be to adjust the feed composition (e.g. if the participant is experiencing an alteration in bowel habit, fibre will either be reduced or increased depending on the content of the current feed). If these steps do not effect an improvement, the total amount of energy provided by the feed will be reduced (temporarily). JEJ feeds will only be stopped completely after discussion with the Healthcare Team.

### Control

Participants randomized to the control group will continue to receive routine clinical care: JEJ feeds will be discontinued on day of discharge, the JEJ tube will remain in place until outpatient review in week six, and prior to discharge the participants will be taught how to flush the JEJ tube daily. Post-discharge dietetic follow-up will involve a routine telephone call and home visit from HENs within the first week of discharge. Additional HEN input will be dictated by nutritional need but will include at least one further contact within the six-week study period.

Following standard practice it may be considered necessary by the healthcare team to re-commence JEJ feeds which we anticipate may be the case in up to 15% of the control group. The criteria for recommencement of the feed will include the following: (1) more than 5% weight loss from baseline or hospital discharge value (visits 1 or 2), (2) clinical assessment by the managing team based on functional status (worsening dysphagia, exhaustion, or anorexia), or (3) oral energy intake reduced to one third of the estimated requirements.

In these situations, JEJ feeds will be restarted according to current practice (if necessary the patient will be readmitted to hospital). The trial participant will continue to be monitored following the trial schedule and the results will be analyzed on both an intention-to-treat and perprotocol basis. The only difference between the intervention and control arms will be the management and administration of the supplementary JEJ feeding for six weeks. All participants will receive clinical reviews following routine care from the multi-disciplinary team (MDT). It is likely that a small number of patients randomized to either group will require home jejunostomy feeding as part of their routine clinical care. These patients will continue feeding in accordance with their planned care. Consideration will be given to withdrawing the participant from the study where there has been major protocol deviation.

### Qualitative protocol

For those participants recruited into the qualitative exploratory study, face-to-face semi-structured interviews, will be conducted. The aim of this part of the study is to explore the experiences of people with upper gastrointestinal cancer and their carers regarding home jejunostomy feeding. Initially, purposive sampling will be adopted to ensure an appropriate selection of cases. The interviews will all follow the same semi-structured format which encourages exploration and discussion of the following areas: experiences of home enteral feeding, ability to comply with the feeding regimen, ability to care for the JEJ tube, impact on activities of daily living, experiences of eating and concerns over oral intake and nutritional status.

This part of the study requires recruitment of a maximum of 20 participants and their carers or partners.

## Outcome measures

### Quantitative data

The primary outcome measures in this pilot study have been chosen to determine whether or not an appropriately powered definitive trial will be possible. To this effect, the primary outcome measures are recruitment and retention rates at six weeks and six months post-baseline. Additional measures to be recorded at discharge, six weeks, three months and six months after baseline focus on the nutritional status and quality of life, specifically (1) nutritional parameters of weight, body mass index, upper arm anthropometry, and grip strength, (2) nutritional intake to include total energy (kcal/d) and protein intake (g/day), contribution of oral intake (food, fluids), oral nutritional supplements, and JEJ feed, (3) postoperative complications, (4) QoL using the European Organization for Research and Treatment of Cancer (EORTC) generic cancer questionnaire (QLQ-C30)[25] and the EORTC disease specific (esophagogastric) questionnaire (QLQ-OG25) [26].

To enable preliminary assessment of cost-effectiveness, QoL using the five domain EuroQol health outcome instrument (EQ-5D) [27] will be measured at three and six-months post-baseline and healthcare costs will be recorded for the duration of the study period. Survival data will also be collected at 12-months post-surgery.

Adverse events and serious adverse events will be defined in accordance with both the Medicines for Human Use (Clinical Trials) Regulations 2004 (SI2004/1031) and the subsequent amendment regulations (SI2006/1938) and ICH-GCP. Expected adverse events associated with the jejunostomy tube are blockage of the tube or inadvertent removal of the tube rendering feeding impossible and skin infection around the tube. Expected adverse events associated with the feed are bloating and diarrhoea.

Due to the nature of the surgery, it is anticipated that up to 50% of the trial participants will experience a serious adverse event prior to discharge from hospital (after randomization, but before any trial treatment has been administered). These adverse events will be recorded for scientific rigor but will not be reportable to the study sponsor. These include death, return to the operating theatre, return to the intensive care unit for re-ventilation, prolonged ventilation (over 72-hours duration) and pneumonia. Table 1 details the planned visit schedule, including acceptability in the timing of visits and measurements. Assessments will either be carried out at the hospital to coincide with routine clinic visits or at home, depending on participant preference.

**Table 1:** Summary of visit schedule and assessed parameters at each time point

**Parameters Assessed: Time Line**										
Pod = post operative day	**VISIT 1**	**VISIT 2**	Week 1	Week 2	Week 3	Week 4	Week 5	**VISIT 3**	**VISIT 4**	**VISIT 5**
	Enrolment (POD -2 wks)	Discharge from hospital (approx POD10)						Week 6	3/12 Clinic	6 / 12 Clinic
**Acceptable Variability**	**+/- 7 days**	**+/- 4 days**						**+/- 7 days**	**+/- 14 days**	
			**Intervention period – home feeding**							
Baseline Characteristics	**X**									
Weight (kg)	**X**	**X**						**X**	**X**	**X**
Height (m)	**X**									
Body mass index (kg / m^2^)	**X**	**X**						**X**	**X**	**X**
Anthropometrics	**X**	**X**						**X**	**X**	**X**
Mid arm circumference										
Anthropometrics	**X**	**X**						**X**	**X**	**X**
TricepSkinfold										
Thickness										
Anthropometrics	**X**	**X**						**X**	**X**	**X**
Grip strength										
Nutritional intake (3 day food diary)		**X**						**X**	**X**	**X**
Post-operative complications		**X**	Collected by Research/Community Dietitian Prospectively						**X**	**X**
Healthcare Resource use	X									**X**
Quality of Life EORTC	**X**	**X**						**X**	**X**	**X**
Quality of Life EQ-5D	**X**							**X**		**X**
Semi-structured interview	End of intervention period									
	Sample of up to 20 trial participants									

The University of Leicester Clinical Trials Unit has developed a trial-specific database. Data will initially be collected onto paper case reports forms and subsequently entered into the electronic database.

### Qualitative data

For those participants also recruited into the qualitative part of the study there will be one interview to take place at the end of the six-week intervention period. Interviews will be conducted in a private room with a member of the research team who has received communication skills and interview training. Interviews should take between 30 and 60 minutes. Following each interview the researcher will make field notes including any observations. This will help to inform reflexive reflection. The interview will be digitally recorded and then transcribed verbatim. Participants will be assigned a false name when the interview is transcribed and the recording will be destroyed when no longer needed for study purposes. To help inform the design of future research in this area, all participants will be asked to provide details of their experiences of being involved in a research trial, in particular the recruitment, randomization, and assessment processes. All participants will be asked on the consent form whether they consent to being contacted at the end of their involvement in the study to complete the questionnaire.

## Data analysis

### Quantitative data

As this is a pilot study and we aim to estimate a number of quantities necessary for the design and delivery of a definitive multi-centre trial, the estimates calculated will be presented along with 95% confidence intervals where appropriate. The proportion of eligible patients who consent to participate in the pilot will be calculated, along with the proportions in each intervention group completing six weeks and six months of assessments. Hospital readmission rates will be presented by intervention group. Variability of the QoL measures will be calculated, both by each group and pooled across both groups.

As it is anticipated that the likely outcome measure for the multi-centre trial will be QoL, a comparison of the QoL measure EORTC QLQ-C30 will be conducted using the Students t-test to compare the mean between the groups to provide a preliminary estimate of the treatment effect. Distribution of the data will be assessed graphically and if the normality assumption is considered to be unreasonable a non-parametric approach will be taken. A secondary analysis, using analysis of covariance (ANCOVA) and regression models will be conducted to adjust for type of surgery (used as a stratification factor in randomization) and baseline value of EORTC QLQ-C30.

The findings of the qualitative research may lead to a different outcome measure being proposed as the primary in the definitive trial and consequently the other QOL measures and other continuous outcomes at six weeks, three months, and six months will be analyzed similarly.

### Qualitative data

A constant comparative approach to the analysis, using the principles of grounded theory [27,28], will be performed on the data. Data analysis will commence following the first two interviews. Following this, in line with the principles of grounded theory and to help focus the data collection, theoretical sampling techniques will be employed and the interview schedule amended as necessary. Data collection will continue until saturation is reached (no new themes are emerging). Similarities or differences between each participant’s experience with regards to a particular topic will be highlighted in the data. Open coding followed by focused coding and theoretical memo writing will lead to the emergence of a coding framework and ultimately theory development. Data organization and retrieval will be managed using the qualitative software package NVivo, QSR International Ltd, Warrington, UK. Use of the software will also assist with methodological auditability.

### Health economics

An economic evaluation will be undertaken comparing the two groups to determine the cost-effectiveness of JEJ feeding. Patient questionnaires will be distributed to collect the cost to the NHS or personal social service and patient or carer cost. The main outcome for the cost utility analysis will be Quality Adjusted Life Years (QALYs) calculated from EQ-5D-3 L using linear interpolation and area under the curve methods. We will test the differences in QALYs calculated from EQ-5D values and EORTC QLQ-C30 measure of quality of life using the method described by McKenzie and van der Pol [29]. Results will be presented as incremental cost-effectiveness ratio (ICER) between the two groups.

### Ethics and dissemination

Approval for the study was granted by the Nottingham Research Ethics Committee (protocol #11/EM/0383) in January 2012 and recruitment commenced in July 2012. The findings will be pertinent to the disciplines of both dietetics and surgery. It is anticipated that the findings will be presented at national meetings such as the British Association of Parenteral and Enteral Nutrition (BAPEN), the Association of Surgeons of Great Britain and Ireland, and international meetings such as the American Gastroenterological Association and the Society for Surgery of the Alimentary Tract. The study findings will be reported in peer reviewed journals of interest to both dietetic and surgical audiences.

We will publish a report of the research findings on both the University of Leicester and the University Hospitals of Leicester NHS Trust websites. We will produce a ‘patient-friendly’ report, produced in close collaboration with the lay members of the steering group. The steering group will be central to the dissemination of results to the wider research community and other users of the health service. It is expected that the Esophageal Patients Association will be involved in the dissemination of the research. We will present the results at the biannual East Midlands Cancer Network meeting and the regional BAPEN meetings. Service users will be informed at local patient support groups and we will also aim to publish results in the national patient support group (PINNT) newsletter.

## Discussion

Currently, there are no guidelines relating to the provision of home feeding after esophagogastric cancer surgery. Policy is determined at a local level with wide heterogeneity in UK practice. This pilot study is an essential precursor for the development of an appropriately powered multi-centre trial and will enable estimates of variability, recruitment and retention rates, and treatment costs, as well as inform choice of appropriate primary outcome for a multi-centre trial. Importantly it will also allow the acceptability of home JEJ to patients to be ascertained. By providing home feeding it is envisaged that more patient care would be delivered locally with a reduced need for hospital readmission for nutritional problems in the first months after surgery. This could represent a cost-effective use of NHS resources. If demonstrated to be beneficial, the findings of a trial assessing the effectiveness of home feeding would be applicable to the 1500 to 2000 patients undergoing esophagectomy or total gastrectomy annually in the UK, potentially saving costs to the NHS and improving the postoperative patient experience.

## Trial status

The study has full ethical and institutional approval and started recruitment in July 2012.

## Abbreviations

ANCOVA: Analysis of Covariance; BAPEN: British Association for Parenteral and Enteral Nutrition; EORTC: European Organisation for Research and Treatment of Cancer; HEN: Home Enteral Nutrition; ICER: Incremental Cost Effectiveness Ratio; ICH-GCP: The International Conference on Harmonisation of Technical Requirements for Registration of Pharmaceuticals for Human Use Guideline for Good Clinical Practice; IWRS: Interactive Web Response System; JEJ: Jejunostomy; MDT: Multi-disciplinary Team; NHS: National Health Service; PINNT: Patients on Intravenous and Nasogastric Nutrition Therapy; QALY: Quality Adjusted Life Year; QoL: Quality of Life; RCT: Randomised Controlled Trial.

## Competing interests

DB and ALT receive departmental grant support from Fresenius-Kabi.

## Authors’ contributions

All authors contributed to the study design, planning and protocol writing. DJB and MB as lead investigators sought research ethics committee approval for the study and coordinate the study on an ongoing basis. VH undertakes all interview studies and will perform the qualitative analysis. RPJ devised the health economics data collection instruments and will perform health economic analysis. ALT and KS provided guidance through the protocol writing, statistical advice and advice about public and patient involvement. All authors will contribute to the analysis and write up. All authors read and approved the final manuscript.
